# New Markers: Urine Xanthine Oxidase and Myeloperoxidase in the Early Detection of Urinary Tract Infection

**DOI:** 10.1155/2014/269362

**Published:** 2014-01-29

**Authors:** Pınar Ciragil, Ergul Belge Kurutas, Meral Miraloglu

**Affiliations:** ^1^Yasamlab Laboratory, Department of Clinical Microbiology, Guzelevler Mahallesi Papatya Sokak Mobilyacılar Sitesi 6, Blok No. 13, Yuregir, 01220 Adana, Turkey; ^2^Sutcu Imam University, Faculty of Medicine, Department of Biochemistry, 46050, Kahramanmaras, Turkey; ^3^Cukurova University, Faculty of Medicine, Department of Microbiology, 01050, Adana, Turkey

## Abstract

*Objectives.* The aim of this study was to evaluate if xanthine oxidase and myeloperoxidase levels quantitation method may alternate routine culture method, which takes more time in the diagnosis of urinary tract infections. *Material and Methods.* Five hundred and forty-nine outpatients who had admitted to Clinic Microbiology Laboratory were included in the study. The microorganisms were identified by using VITEK System. The urine specimens that were negative from the quantitative urine culture were used as controls. The activities of MPO and XO in spot urine were measured by spectrophotometric method. *Results.* Through the urine cultures, 167 bacteria were isolated from 163 urine specimens; 386 cultures yielded no bacterial growth. *E. coli* was the most frequent pathogen. In infection with *E. coli* both XO and MPO levels were increased the most. The sensitivity, specificity, positive predictive value, and negative predictive value for XO were 100%, 100%, 100%, and 100%, respectively. These values for MPO were 87%, 100%, 100%, and 94%, respectively. *Conclusion.* These data obtained suggest that urine XO and MPO levels may be new markers in the early detection of UTI.

## 1. Introduction

Urinary tract infections (UTIs) are the most common of all bacterial infections affecting human beings throughout its life span. UTI occurs in all age groups from neonates to the geriatric patients. UTIs range from an asymptomatic infection to acute pyelonephritis with gram-negative septicemia to symptomatic bacteriuria. There can be many complications of urinary tract infections, including dehydration, sepsis, kidney failure, and death. But if treated early and adequately, the prognosis is good for most patients with a UTI. Although culture method is the gold standard in diagnosis of UTI, unfortunately it is time and money consuming [1].

Reactive oxygen species (ROS) could be generated by a variety of reasons at the cellular levels. An important source of ROS is known to be xanthine oxidase (XO) that could be formed from xanthine dehydrogenase either reversibly (via oxidation or blockage of its thiol groups) or irreversibly (via limited proteolysis) under pathological conditions [2]. The elevated tissue or serum XO activities are thought to be responsible for mechanism of several pathological conditions including alcoholism and smoking [3, 4].

Myeloperoxidase (MPO) is an inflammatory marker. Studies indicated that MPO holds a central role in microbial killing in humans and animals. Upregulation of MPO gene expression in activated phagocytes would seem consistent with the presumed antimicrobial function of the enzyme in these cells. Recent investigations revealed a crucial role of MPO in chronic, nonmicrobial inflammatory processes such as neurodegenerative disease and atherosclerosis [5].

To our knowledge none of the previous researchers investigated both of the XO and MPO according to each bacteria strain in infected urine samples. Therefore in determination of UTI, the quantitation method of XO and MPO levels had been suggested in this study. The sensitivity and specificity of a test may not be used for to estimate the probability of disease in a patient. So predictive values may be more useful measures of diagnostic accuracy. So we want to determine the sensitivity, specificity, positive predictive value (PPV), and negative predictive value (NPV) of XO and MPO, if these markers were utilizable in diagnosis of UTI. Also, this study was planned to determine if the measurement of XO and MPO levels may alternate to the routine urine culture method which is time and money consuming in UTI.

## 2. Material and Methods

A total of 549 urine specimens were collected and analyzed between March 2006 and May 2007 from outpatients who were clinically suspected of UTI. Inclusion criteria were dysuria, frequency, urgency, and abdominal/flank pain with or without fever. Specimens of patients who were receiving antibiotic therapy were excluded from the study. The specimens were collected at location in city of Kahramanmaras, Turkey, The Laboratory of Clinical Microbiology, Faculty of Medicine, Kahramanmaras Sutcu Imam University. The outpatients ranged in age from 1 to 75; 278 of them were females and 271 were males. The spot urine samples were collected using the clean-catch, midstream urine specimen collection method in sterile plastic containers (Kayline Plastics, The barton, South Australia, 5031) for adults and with application of a sterile plastic adhesive bag for pediatric patients. The samples were transported to the laboratory within 30 minutes for microbiologic and biochemical analyses. The study was approved by the local ethics committee of the institution.

### 2.1. Microbiologic Analyses

The blood agar (BA) (BD, BBL, NJ, USA) and MacConkey (MAC) (BD, BBL) were received as ready-to-use agar plates. All plates were randomly inoculated in parallel by the same person using a 0.01 mL calibrated loop. They were then incubated aerobically at 37°C for 18–24 h. Each new lot of the media was tested for sterility, ability to support growth, and biochemical response using control strains from the American Type Culture Collection.

Growth of one or two possible urinary pathogens at a concentration of ≥10^5^ colony forming unit/mL (cfu/mL) was considered significant and those isolates were identified. Urinary isolates from positive cultures were identified by using VITEK System (Bio Mérioux, France) and conventional biochemical methods. The urine specimens that were negative from the quantitative urine culture were used as controls.

### 2.2. Biochemical Analyses

#### 2.2.1. Urine Samples

The spot urine samples of subjects were diluted with 1 : 50 serum physiologic (0.9% NaCI) for biochemical analysis. To control the urine concentration, data were normalized to urine creatinine concentration. Urinary creatinine levels were measured in spot urine samples by Dade Behring Dimension RXL autoanalyzer (Germany). Therefore, the results of XO and MPO were given over creatinine.

#### 2.2.2. Measurement of MPO Activity

MPO activity was determined by the O-dianisidine method [6]. The assay mixture, in a cuvette with a path length of 1 cm, contained 0.3 mL 0.1 M phosphate buffer (pH), 0.3 mL 0.01 M H_2_O_2_, 0.5 mL 0.02 M O-dianisidine (freshly prepared) in deionized water, and 10 mL urine sample in a final volume of 3 mL. The urine sample was added last and the change in absorbance at 460 nm was followed for 10 min. All measurements were carried out in duplicate. One unit of MPO is defined as that giving an increase in absorbance of 0.001 per min and specific activity is given as U/L.

#### 2.2.3. Measurement of XO Activity

Xanthine oxidase (XO) activity was assayed as described by Prajda and Weber [7]. The reaction mixture containing 0.2 mL sample was diluted to 1 mL with phosphate buffer and incubated for 5 min at 37°C. The reaction was started by adding 0.1 mL xanthine and kept at 37°C for 20 min. The reaction was terminated by the addition of 0.5 mL ice-cold perchloric acid (10%). After 10 min, 2.5 mL distilled water was added to it and the mixture was centrifuged at 4000 rpm for 10 min. The absorbance of the urine samples was read at 290 nm. The activity of XO is expressed as U/L.

### 2.3. Statistical Analysis

Statistical analysis was performed by SPSS, a commercially available statistics software package. Group comparisons (between control and patient groups) were performed using the *t*-test. A *P* < 0.05 was considered to be statistically significant. We also estimated the sensitivity, specificity, PPV, NPV, and accuracy of XO and MPO for all samples.

## 3. Results

Through the urine cultures, 167 bacteria were isolated from 163 urine specimens; no microorganism was found in the remaining 386 specimen. A urine culture with ≥10^5^ cfu/mL was considered to be positive and a culture with ≤10^4^ cfu/mL was considered to be negative. The single strain microorganisms were identified in 159, and two microorganisms were identified in four positive urine cultures, which were as follows: 87 (52.09%) *E. coli*, 30 (17.96%) *K. pneumoniae*, 13 (7.8%) *S. saprophyticus*, 2 (1.2%) *C. albicans*, 12 (7.2%) *Candida* spp., 8 (4.8%) *E. faecalis*, 8 (4.8%) *E. faecium*, 3 (1.8%) *P. aeruginosa*, 2 (1.2%) *S. agalactiae*, 1 (0.6%) *P. mirabilis*, and 1 (0.6%) *E. cloacae*. The two strain microorganisms were identified in four positive urine cultures and were *E. coli + P. mirabilis*, *K. pneumoniae + E. faecium*, *S. saprophyticus + E. faecalis*, and *E. faecium + Candida *spp.

The XO and MPO levels in the normal and the infected urines are presented in Tables 1 and 2. In all infected urines, the level of XO activity was above 2000 U/L except in fourteen urine harbouring *Candida* spp. and *Candida albicans* and one in *P. mirabilis*. The activity of XO levels was the highest (10820.2 ± 1543.7 U/L) in the samples which were containing *E. coli*. In the 386 sterile urine samples, the XO enzyme level was 104.57 U/L and XO activity ranged from 17 to 271 U/L. It can be seen from the table that in the urines containing pathogenic bacteria, the highest XO activity was found in urines containing *E. coli* and the lowest activity in urines containing *Candida* spp. and *P. mirabilis.* Urine specimens containing two strain growth cultures were also studied. In all these urines XO activity could be related to the total concentration of bacteria.

MPO levels were 414 U/L in sterile urine samples. While also MPO level was the highest (1546 U/L) especially in infected urines with *E. coli*, it was the lowest in infected urines with *S. agalactiae* and *E. cloacae*.

Also, both of enzyme levels increased in UTI as shown in Tables 1 and 2. Both biomarkers (XO and MPO) showed high sensitivity, specificity, PPV, and NPV as shown in Table 3.

## 4. Discussion

To our knowledge, this is the first study to carry out the determination of the link between levels of both enzymes levels and bacteria strains in urine samples of patients with UTI. In this study, we showed significantly elevated levels of XO and MPO in patients with UTI than in healthy controls. Increases in XO and MPO may play the role in aetiopathogenesis of UTI. Some enzymes which have been most extensively studied in the urine as markers for urinary tract infection are lactic dehydrogenase, glutamic oxaloacetic transaminase, alkaline phosphatase, *β*-glucuronidase, catalase, and superoxide dismutase [8]. In view of the contribution of tissue-derived enzymes to the urinary enzymatic activities, the use of a urinary enzymic marker for the detection UTI would require that urinary enzymatic activity be derived from the pathogenic bacteria only, with exclusion of tissues sources. For this reason we have chosen XO and MPO enzymes.

XO enzyme is confined in man mainly to the liver; a significant but lower activity has been found also in jejunal mucosa and colostrum; on the other hand, the kidney, prostate, and blood elements, as well as normal serum, are virtually devoid of XO activity [9–14]. Indeed, we showed that urine XO activity was increased in patients with UTI, conforming to previously reported results, indicating that plasma XO is transferred to the urine, probably due to its low molecular weight. Thus, urinary XO activity seems to constitute a satisfactory indication of the presence of UTI. Indeed, in the present study, urinary XO activity was shown to relate specifically to pathogenic urinary bacteria. Increased activity is being present only in the urines containing bacteria in amounts greater than 10^5^ per mL. Besides this the sensitivity (100%) and specificity (100%) of XO were good enough to use in diagnosis of UTI. Preliminary studies with the pathogenic bacteria isolated from the infected urines have shown that cultures of these bacteria displayed XO activity. Studies are in progress to characterise the bacterial enzymes involved in the degradation of hypoxanthine and to quantitative methods of urinary XO estimation.

Many authors demonstrated the presence of MPO containing cells as well as MPO protein and activity in many renal diseases [15, 16]. The adherence of neutrophils to the glomerular basement membrane and the degradation of the basement membrane by oxidants at sites of attachment [17] pointed toward a direct involvement of MPO. In vivo experiments (i.e., perfusion with MPO followed by nontoxic concentrations of hydrogen peroxide and chloride ions) revealed MPO-mediated glomerular disease resulting in glomerular morphologic changes, endothelial and mesangial cell injury, activation of platelets, and subsequent proliferative responses mimicking inflammatory and proliferative glomerulonephritis in humans [18]. We detected MPO activity increased in the urine patients with UTI detected. We also determined high sensitivity (87%) and specificity (100%) values for MPO activity. Steinhoff et al. showed that MPO activity is increased in the urine as a noninvasive diagnostic marker of UTI in patients with renal transplantation. They mentioned that urinary MPO showed linear correlation to number of leukocytes in urine and renal biopsy is confirmatory than diagnosis in this group of patients [19]. Hillegass et al. demonstrated that MPO activity was significantly greater than that from the control from the inflamed kidney tissue [20]. Fraser et al. reported that in the urine of men with *Neisseria gonorrhoeae* or *Chlamydia trachomatis* infections and those with urethritis showed MPO levels to be nondiscriminatory; however neutrophil elastase levels were significantly elevated in patients with proven infection or urethritis or both [21].

The microbiology laboratory plays a fundamental role in the diagnosis, therapy, and monitoring of UTI. Rapid identification of the etiological agent can provide the clinician with important information regarding the appropriate choice of an antibiotic even before the results of susceptibility tests are available. Performing urine cultures for the diagnosis of UTI is a significant part of the workload of most clinical microbiology laboratories. Since urine samples are the most frequently analyzed specimens in microbiology laboratories, but only a minor percentage of isolates is significant, the evaluation of clinically insignificant isolates is a waste of resources; so rapid identification of etiological agents enables the clinician to choose the appropriate antibiotic, even before the results of susceptibility testing are available. In the present study, infected urine samples in the urine levels of XO and MPO were higher than sterile urine samples. *E. coli* is responsible for most of the UTIs in patients [1]. *E. coli* was the predominant agent in this study and the XO and MPO enzyme levels are all increased in urine samples which were infected with *E. coli* (Table 1). Increased XO activity in infected urine samples especially in urines infected with *E. coli* constitutes a satisfactory indication of the presence of UTI in our study. We also found the lowest activity in urine containing *P. mirabilis* and *Candida* species, so this result is compatible with the study of Al-Khalidi and Chaglassian [13]. MPO enzyme levels are also increased in infected urine samples (Table 1). Although the specificity of this test via bacterial infection may be doubted, Steinhoff et al. also discussed the measurement of urinary MPO levels as a possible noninvasive diagnostic marker for renal graft monitoring [22].

Sackett et al. mentioned that a test with a high specificity is useful for “ruling in” a disease if a person tests positive [23]. The specificity of XO and MPO levels in our study was 100% and 100% respectively. So these markers will be utilizable in early diagnosis of UTI. The sensitivity and NPV of XO are higher than MPO, so XO may be a better marker than MPO for the diagnosis of UTI to begin the antibiotherapy without delay of culture results.

## 5. Conclusion

This recent study shows that in patients suspected of UTI, by studying these enzymes and beginning the antibiotherapy before culture test results may be time consuming for the patient and the doctor.

This present study is the first to report significantly increased XO and MPO activities in urine derived from subjects with UTI, and they are tempting to speculate that urinary XO and MPO could be directly involved in the pathogenesis. Further studies should be directed towards evaluating their use in the diagnosis of UTI.

## Figures and Tables

**Table 1:** Xanthine oxidase and myeloperoxidase activities in sterile and infected urines with single bacteria.

Urine samples	XO (U/L)		MPO (U/L)	
	Mean ± SD	Min–max	Mean ± SD	Min–max
Sterile (386)	104.57 ± 49.28	17–271	414.09 ± 93.31	156–745
Infected urines (163)				
*Escherichia coli* (87)	10820.2 ± 1543.7*	5780–15370	1025.8 ± 251.3*	510–1546
*K. pneumoniae* (30)	3108.4 ± 596.7*	1800–4235	522.0 ± 134.4*	300–780
*S. saprophyticus* (13)	4370.0 ± 1492.5*	2340–7800	459.5 ± 138.5*	289–756
*E. faecium* (8)	3045.7 ± 824.9*	2000–4350	475.0 ± 86.3*	310–560
*E. faecalis* (8)	3431.2 ± 812.7*	2100–4600	407.6 ± 88.7*	298–524
*P. aeruginosa* (3)	6146.0 ± 534.1*	5700–6738	624.3 ± 112.1*	520–743
*E. cloacae* (1)	4300**		345**	
*S. agalactiae* (2)	2850.0 ± 494.9*	2500–3200	390.0 ± 32.5	367–413
*P. mirabilis* (1)	1543**		413**	
*Candida* spp. (12)	1807.9 ± 647.8*	1000–2700	649.0 ± 102.9*	488–760
*Candida albicans* (2)	1930.0 ± 523.2*	1560–2300	480.0 ± 84.8	420–540

Numbers in parentheses indicate number of patients examined.

*P < 0.05.

**Statistical test was not applied for single samples.

**Table 2:** Xanthine oxidase and myeloperoxidase activities in sterile and infected urines with binary growth.

Urine samples	XO (U/L)	MPO (U/L)
Sterile (386)	104.57 ± 49.28	414.09 ± 93.31
*E. coli + P*. *mirabilis* (1)	16100*	1470*
*K. pneumoniae + E. faecium* (1)	5700*	654*
*S. saprophyticus + E. faecalis* (1)	5980*	760*
*E. faecium + Candida* spp. (1)	4900*	657*

*Statistical test was not applied for single samples.

**Table 3:** The sensitivity, specificity, predictive values, and accuracy of XO and MPO in diagnosis of UTI.

Markers	Sensitivity	Specificity	PPV	NPV	Accuracy
XO	100%	100%	100%	100%	100%
MPO	87%	100%	100%	94%	95%
